# Metabolite Profiling Revealed That a Gardening Activity Program Improves Cognitive Ability Correlated with BDNF Levels and Serotonin Metabolism in the Elderly

**DOI:** 10.3390/ijerph17020541

**Published:** 2020-01-15

**Authors:** Sin-Ae Park, Su Young Son, A-Young Lee, Hee-Geun Park, Wang-Lok Lee, Choong Hwan Lee

**Affiliations:** 1 Department of Environmental Health Science, Konkuk University, Seoul 05029, Korea; danapre@nate.com; 2 Department of Bioscience and Biotechnology, Konkuk University, Seoul 05029, Korea; syson119@naver.com; 3 Sport Science Center in Daejeon, Daejeon 34134, Korea; exepre@cnu.ac.kr; 4 Department of Sport Science, Chungnam National University, Daejeon 34134, Korea; leewl@cnu.ac.kr; 5 Research Institute for Bioactive-Metabolome Network, Konkuk University, Seoul 05029, Korea

**Keywords:** tryptophan metabolism, gardening, horticultural therapy, brain-derived neurotrophic factor

## Abstract

Metabolomics is useful for evaluating the fundamental mechanisms of improvements in the health functions of the elderly. Additionally, gardening intervention as a regular physical activity for the elderly maintained and improved physical, psychology, cognitive, and social health. This study was conducted to determine whether the cognitive ability of the elderly is affected by participating in a gardening activity program as a physical activity with a metabolomic potential biomarker. The gardening program was designed as a low to moderate intensity physical activity for the elderly. Serum metabolites resulting from gardening were subjected to metabolite profiling using gas chromatography time-of-flight mass spectrometry and ultra-high-performance liquid chromatography-linear trap quadruple-orbitrap-mass spectrometry followed by multivariate analyses. The partial least squares-discriminant analysis showed distinct clustering patterns among the control, non-gardening, and gardening groups. According to the pathway analysis, tryptophan metabolism including tryptophan, kynurenine, and serotonin showed significantly distinctive metabolites in the gardening group. Brain-derived neurotrophic factor levels (BDNF) in the gardening group were significantly increased after the gardening program. Correlation map analysis showed that the relative levels of tryptophan metabolites were positively correlated with BDNF. Our results show that tryptophan, kynurenine, and serotonin may be useful as metabolic biomarkers for improved cognitive ability by the gardening intervention.

## 1. Introduction

The elderly lose the capacity to maintain health functions such as physical, psychology, cognitive, and social functions when aging [1]. Particularly, cognitive aging causes defects in memory, a decline in learning and intellect, and cognitive disorder with loss of hippocampal function and loss of brain volume in the cerebrum [2,3]. Additionally, cognitive aging occurs in neurodegenerative diseases such as vascular dementia, Alzheimer’s disease, and Parkinson’s disease [4]. The hippocampus is important for recent memory formation in the temporal lobe of the brain. The volume and weight of the hippocampus decrease by 1%–2% each year during aging in the elderly [5,6,7]. A larger hippocampal size leads to a better memory and cognitive ability [8].

A study showed most elderly people more than 10 countries—UK, France USA, Canada, China, Japan, etc.—and one EU-wide spend approximately for 65%–80% of each day conducting sedentary activities (e.g., watching TV, playing card games, lying on a bed, etc.) [9]. Decreases in physical activity because of a sedentary lifestyle accelerate the aging process [10]. This age-associated loss of heath function can be prevented by physical activity [11], which may help improve cognitive health [12,13,14]. Studies have shown that increases in hippocampal size, gray matter, and white matter volumes, and brain-derived neurotrophic factor (BDNF) in the human brain are typical responses that occur during cognitive function (e.g., short-term memory, visual perception memory, and learning) of individuals participating in physical activity [12,13,14,15,16,17]. Patients with Alzheimer’s disease have significantly lower serum BDNF levels compared to healthy controls [18]. The nerve growth factor plays an important role in neurogenesis and has been implicated in several molecular processes in the central nervous system [19]. The levels of the nerve growth factors can be quantified and represent the status of cognitive health in humans. Commonly measured nerve growth factors in clinical studies include BDNF, vascular endothelial growth factor (VEGF), and platelet-derived growth factor (PDGF). BDNF is present in the hippocampus and its levels are stimulated by physical activity, which leads to the growth of new nerve cells in the brain for learning and long-term memory [20]. VEGF and PDGF are also affected by physical activity, but show a greater association with vascular formation, permeability, and connective tissue changes. These molecules are up-regulated after exercise and can promote cell proliferation and growth as well as neuronal development and function [21].

Gardening activity is regarded as a low to moderately intense physical activity [22,23,24], while weight-bearing exercises use all muscles in the body [25,26] of the elderly. Gardening intervention conducted as a regular physical activity maintained and improved the physical, psychology, cognitive, and social health of the elderly [27,28,29,30,31,32]. A 15-session gardening intervention significantly improved physical function (e.g., muscle mass, aerobic endurance, blood pressure, cholesterol, hand dexterity), immune function (e.g., oxidative stress, inflammation), and cognitive function and decreased depression in elderly women [28,29]. Additionally, a study showed that touching soil increased the secretion of serotonin, which is a hormone that generates antidepressant effects on behavior [33]. Aging also reduces the levels of serotonin [34] and its receptors [35], which inhibits its antidepressant effects. A 10-session gardening intervention improved muscle strength in the upper and lower limbs as well as flexibility, agility, aerobic endurance, balance, and decreased stress, which was confirmed by lower levels of cortisol in elderly adults [27]. Park et al. [28] investigated the effects of gardening intervention on neurological functions in serum analysis. They reported that a 20-min gardening activity as a short-term physical activity significantly increased the levels of brain nerve growth factors (e.g., BDNF, PDGF) that are related to cognitive ability in the elderly. However, these previous investigations did not explore the underlying processes involved in such improvements. How these improvements are triggered and moderated by a series of mechanistic processes is not well-understood.

Metabolomics is the comprehensive analysis of the levels of metabolites in biological systems such as cells, tissues, and organs, which can be used to evaluate the fundamental mechanisms surrounding improvements in the health functions of the elderly [36,37]. Metabolic profiling provides a biochemical blueprint of physiological alterations in response to stimuli, which can also help identify key compounds involved in specific changes. These key compounds can be further analyzed to identify correlations among various phenotypes. Examination of the correlations between key compounds and changes in their levels can reveal the relationships between metabolism-related factors and suggest a mode of action [38,39,40]. In this study, we investigated the effect of a gardening activity program in the elderly by metabolite profiling in serum. Furthermore, key metabolites with gardening activity efficacy were correlated with various phenotypes such as body composition, cognitive ability, and physical activity.

Therefore, this study was conducted to identify the mechanistic processes and key metabolites of health improvements following a gardening program as physical activity for improving the cognitive ability of the elderly using metabolic profiling techniques.

## 2. Materials and Methods

### 2.1. Recruitment and Experimental Design

To recruit the elderly at a senior welfare center for this study, a flyer with a description of the study purpose, gardening intervention, and health measurements was posted on the homepage of the Seoul Association of Senior Welfare Center. The senior welfare center located in Eunpyeong-gu, Seoul, South Korea was selected for this study. Forty elderly people aged 65 years or older accommodated by this center decided to participate in the study and signed consent forms for the study. This study featured a quasi-experimental design with a nonequivalent control group. Twenty elderly people participated in the 24-session gardening program. Another 20 elderly people comprised the non-gardening group. This study was conducted over a total of 12 weeks from May to July 2018. The study was approved by the institutional review board of Konkuk University (7001355-201804-HR-238).

### 2.2. Gardening Program

The 24-session gardening program was developed as low-intensity to moderate-intensity physical activities to improve cognitive function in the elderly. Previously reported exercise intensity data for various gardening activities performed by the elderly were used to select gardening activities [22,41,42]. The program involved twice-weekly sessions for an average duration of 60 min per session. The selected gardening activities of the program were garden design and planning, planting transplants, sowing seeds, cutting, garden maintenance, and hydroponics (Table 1). Each participant was provided with a separate garden plot of a 1.2 m (W) × 1.8 m (L).

### 2.3. Health Assessments

Health assessments were performed for subjects in both groups before and after participating in the 24-session gardening program. Growth factor levels are related to cognitive function such as BDNF, VEGF, PDGF, and physical health conditions. Additionally, physical functional ability, hand functional ability, and cognitive ability were assessed.

For subjects from both groups, 10 mL of blood was collected to analyze growth factor levels before and after the gardening program. Before blood sampling, the subjects were asked to fast for 9 h. Sampling was conducted in the early morning between 7:00 and 9:00 by a certified nurse visiting on site.

To assess physical functional ability, we used the Senior Fitness Test [44], which measures physiological parameters using functional movement tasks such as standing, bending, lifting, reaching, and walking. This test meets scientific standards for validity and reliability [45] and has been developed as a tool for evaluating the functional fitness performance of elderly adults [44]. The age-based and gender-based norms in the test were determined in more than 7000 elderly adults in 21 states of the US and published for each test item. There are six assessment items in the Senior Fitness Test [42] including the chair stand test, arm curl test, chair sit and reach test, back scratch test, 2-min step test, and the 2.45 m up and go test. The subjects were provided with an oral explanation and demonstration of each test, and then allowed to practice the test motions before starting the evaluation.

To assess hand function ability, we used a digital grip dynamometer (KS-301, Lavisen, Inc., Namyangju-si, Korea), Jamar hydraulic pinch gauge (749805, Sammons Preston, Inc., Warrenville, IL, USA), and grooved pegboard (32025, Lafayette, Inc., Lafayette, CO, USA) to measure grip strength, pinch force, and hand dexterity, respectively. Grip strength and pinch force were measured on the dominant hand in triplicate, while hand dexterity was measured in duplicate. By definition, hand dexterity (i.e., fine motor skill) is the ability to coordinate small muscle movements that typically involve synchronization of the hands and fingers with the eyes [46,47].

To assess cognitive ability, we used the Korean Mini Mental State Examination [48] to measure disorientation of time, disorientation of place, memory, attention and calculation, memory recall, language, and composition of time and space. The scores from these subscales were summed to a total score ranging from 0 to 30. A total score of ≥24, 18–23, and ≤17 each indicates normal ability, mild cognitive impairment, and severe cognitive impairment, respectively. The Cronbach’s *a* of this instrument is 0.86 [48].

At the beginning of the study, demographic information such as age, gender, physical activity level, education level, marital status, family size, and monthly income was obtained via a questionnaire completed by subjects in both groups. We used the International Physical Activity Questionnaire-Short Form [49] to determine the duration and exercise intensity of daily physical activities of the subjects during the seven-day period prior to the study. The daily physical activities were self-reported in units of the metabolic equivalent of a task (MET). A MET-min was computed by multiplying the MET score by the number of minutes the activity was performed. Additionally, body composition values such as body weight (kilograms), fat mass (grams), lean mass (grams), and percent fat (%) were measured using a body fat analyzer (ioi353, Jawon Medical, Gyeongsan, Korea). Height was measured using an anthropometer (Ok7979, Samhwa, Seoul, Korea).

### 2.4. Sample for Analyzing Brain Nerve Growth Factors

To measure changes in the brain nerve growth factors, the collected blood samples (Section 2.3) were collected and stored in vacutainers packed in ice and transferred to a laboratory for analysis. The blood was centrifuged and serum was stored in microcentrifuge tubes (Eppendorf, Hamburg, Germany) in a freezer at −80 °C. Sandwich enzyme-linked immunosorbent assay (ELISA) kits were used to measure BDNF, PDGF, and VEGF (AbCAM, Cambridge, UK), according to the manufacturer’s instructions. Readings were performed using a microplate reader (Bio-Rad, Hercules, CA, USA) adjusted to a wavelength of 490 nm.

### 2.5. Sample Preparation for Metabolomics Study

Each 200-μL human serum sample was extracted with a solution of cold methanol (1 mL) and 10 μL of internal standard solution (2-chloro-phenylalanine, 1 mg/mL in water) using an MM400 mixer mill (Retsch^®^, Haan, Germany) at a frequency of 30 Hz for 10 min, which is followed by sonication for 10 min. After centrifugation for 10 min at 12,000 rpm and 4 °C (Gyrozen 1730R, Gyrozen Inc., Daejeon, Korea), the supernatant was filtered through a 0.2-μm polytetrafluoroethylene (PTFE) filter (Chromdisc, Daegu, Korea) and evaporated using a speed vacuum concentrator (Modulspin 31, Biotron, Seoul, Korea). The final concentration of each sample was adjusted to 10 mg/mL for mass spectrometry (MS) analysis.

### 2.6. GC-TOF-MS Analysis

Gas chromatography time-of-flight mass spectrometry (GC-TOF-MS) analysis was performed as previously described by Jung et al. [50]. For analysis, all dried samples were oximated with 50 μL of methoxyamine hydrochloride (20 mg/mL in pyridine) for 90 min at 30 °C and silylated with 50 μL of N-methyl-N-(trimethylsilyl) trifluoroacetamide for 30 min at 37 °C. The derivatized samples were analyzed on an Agilent 7890A GC system (Santa Clara, CA, USA) coupled with an Agilent 7693 auto-sampler and Pegasus^®^ HT TOF MS (LECO Corp., St. Joseph, MI, USA). An Rtx-5MS column (30 m × 0.25 mm, 0.25-μm particle size, Restek Corp., St. Joseph, MI, USA) was used at a constant flow of 1.5 mL/min with helium used as the carrier gas. Next, 1 μL of the derivatized samples were injected into the GC in a splitless mode. The GC oven temperature was asset to 75 °C for 2 min, and then increased by 15 °C/min to 300 °C with a 3-min hold time as the final temperature. The mass data collection rate was set to 10 scans/s over a scan range of 50–1000 *m/z* followed by −70 eV of an electron ionization mode. The front inlet and transfer line temperatures were set to 250 °C and 240 °C, respectively.

### 2.7. UHPLC-LTQ-Orbitrap-MS Analysis

Ultrahigh performance liquid chromatography (UHPLC) was performed on a Vanquish binary pump H system (Thermo Fisher Scientific, Waltham, MA, USA) coupled with an auto-sampler and column compartment. Chromatographic separation was carried out on Phenomenex KINETEX^®^ C18 column (100 mm × 2.1 mm, 1.7 μm particle size, Torrance, CA, USA) and the injection volume was 5 μL. The column temperature was set to 40 °C and the flow rate was 0.3 mL/min. The mobile phase consisted of 0.1% formic acid in water (Solvent A) and 0.1% formic acid in acetonitrile (Solvent B). The gradient parameters were set as follows: 5% solvent B was maintained initially for 1 min followed by a linear increase to 100% solvent B over 9 min and then sustained at 100% solvent B for 1 min with a gradual decrease to 5% solvent B over 3 min. The total run time was 14 min. The MS data were collected in the range of 100–1000 *m/z* (under a negative-ion and positive-ion mode) using an Orbitrap Velos ProTM system, which is combined with an ion trap mass spectrometer (Thermo Fisher Scientific) coupled with a Heated Electrospray Ionization (HESI-II) probe. The probe heater and capillary temperatures were set to 300 °C and 350 °C, respectively. The capillary voltage was set to 3.7 kV in a positive mode (negative mode, 2.5 kV). Leucine encephalin was utilized as reference lock mass (*m/z* 554.2615).

### 2.8. Data Analysis

A paired *t*-test was used to compare growth factor levels and physical health conditions such as physical functional ability and hand function ability measured before and after the gardening program in both groups. An independent *t*-test was used to compare cognitive ability. A chi-squared test was used to compare age, sex, body composition, physical activity level, family size, education level, marital status, and current disease in the subjects of both groups. Statistical analyses were performed using SPSS 24 software (SPSS, Inc., Chicago, IL, USA). *p* < 0.05 was considered to indicate statistical significance.

MS data processing and multivariate statistical analysis were conducted as described in our previous study [50]. GC-TOF-MS data were collected and converted into netCDF (*.cdf) format using LECO ChromaTOF software (version 4.44, LECO Corp., St. Joseph, MI, USA). UHPLC-LTQ-Orbitrap-MS data were acquired with Xcalibur software (version 2.00, Thermo Fisher Scientific, Waltham, MA, USA). Raw data were converted to a netCDF (*.cdf) format using Xcalibur software. After conversion, retention time correction, peak detection, peak intensity normalization, and accurate masses were determined using MetAlign software (RIKILT-Institute of Food Safety, Wageningen, the Netherlands). The alignment data were exported to Excel files (Microsoft, Redmond, WA, USA). Multivariate statistical analyses were conducted using SIMCA-P+ software (version 12.0, Umetrics, Umea, Sweden). Principal component analysis (PCA) and partial least squares discrimination analysis (PLS-DA) were preformed to compare different metabolites among experimental groups including in the control, non-gardening and gardening groups. The significance of the partial least squares-discriminant analysis (PLS-DA) model was defined by analysis of variance testing of cross-validated predictive residuals (CV-ANOVA) in the SIMCA-P+ program. Discriminative variables were selected based on variable importance in the projection (VIP) value of the PLS-DA. The selected metabolites obtained from GC-TOF-MS and UHPLC-LTQ-Orbitrap-MS were tentatively identified based on various data comparing their retention time (min), mass spectrum (*m/z*), an MS^n^ fragment pattern with those for standard compounds analyzed under identical conditions, and various, available databases including the Human Metabolome Database (HMDB, http://www.hmdb.ca/), the National Institute of Standards and Technology (NIST) database (Version 2.0, 2001, FairCom, Gaithersburg, MD, USA), Wiley 9, in house libraries, and published papers [51]. Significant differences were evaluated by analysis of variance (ANOVA) and Student’ *t*-test coupled with the Pearson’s correlation coefficient between metabolites and the corresponding phenotype using Predictive Analytics SoftWare (PASW) Statistics 18 software (SPSS Inc., Chicago, IL, USA).

### 2.9. Quantification of Selected Metabolites for Validation

Quantification was performed by GC-TOF-MS analysis to confirm the metabolomics analysis results. Standard compounds were acquired for important metabolites such as lactic acid, tryptophan, kynurenine, and serotonin from the experimental groups including in control, non-gardening, and gardening subjects. The standard compounds were serially diluted as follows: 19.53 μg/mL for lactic acid, 31.25 μg/mL for tryptophan, 15.63 μg/mL for kynurenine, and 7.81 μg/mL for serotonin. Standard compound concentrations were determined to prepare the standard curve, which was coupled with the corresponding regression equation from GC-TOF-MS analysis.

## 3. Results

### 3.1. Demographic Characteristics

The elderly who participated in the gardening program were an average age of 71.8 ± 4.8 years (10 males and 10 females) while those in the control group were an average age of 75.9 ± 5.3 years (four males and 16 females). Age and gender significantly differed between the two groups. The education level of the gardening group was higher than that of the control group (Table 2). In the other surveyed variables, no significant differences were observed (e.g., body composition, physical activity level, family size, marital status, and current diseases, Table 2).

### 3.2. Health Assessments

The elderly people in the gardening group showed significant improvements in BDNF levels and cognitive ability in cognitive health compared to those in the non-gardening group. The elderly people in the 24-session gardening program showed a significant increase in BDNF levels, while the non-gardening group showed a significant decrease in BDNF levels (*p* = 0.047, Table S1). The Korean Mini-Mental State Examination scores of the gardening group were significantly higher than those of the non-gardening group after participating in the gardening program (*p* = 0.003) (Table 3). Moreover, the elderly in the gardening program exhibited a significant improvement in hand dexterity with a decrease in task time (s) 80.6 ± 15.2 to 76.9 ± 13.6 (*p* = 0.024) (Table 4). There were no significant differences in the scores of all Senior Fitness Test items before and after the gardening program in both groups (Table 5).

### 3.3. GC-TOF-MS and UHPLC-LTQ-Orbitrap-MS Analysis for Serum Metabolomes

**Figure 1:**
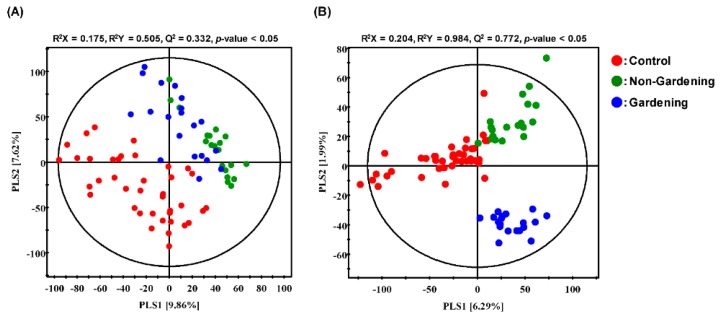
Partial least squares-discriminant analysis (PLS-DA) (**A**,**B**) score plots of the serum sample from control, non-gardening, and gardening group using data from Gas chromatography time-of-flight mass spectrometry (GC-TOF-MS) (**A**) and Ultrahigh-performance liquid chromatography Linear-ion-trap Orbitrap mass spectrometry (UHPLC-LTQ-Orbitrap-MS) (**B**) analysis.

To identify significantly different metabolites among the control, non-gardening, and gardening groups, we performed comprehensive metabolite profiling of serum samples using GC-TOF-MS and UHPLC-LTQ-Orbitrap-MS coupled with multivariate analysis including the unsupervised PCA as well as supervised PLS-DA. The PLS-DA score plot derived from GC-TOF-MS data showed a clearly distinct pattern between the control group and other groups along with PLS1 (9.86%) and PLS2 (7.62%), while the non-gardening group and gardening group were not clearly different from each other (Figure 1A). The statistical parameters of PLS-DA models were evaluated by R^2^X (0.175), R^2^Y (0.505), Q^2^ (0.332), and the *p*-value (<0.05), which indicates the prediction accuracy, fitness, cross-validation analysis, and model validation, as shown in the figures. However, the unsupervised PCA score plots showed no differences (Figure S1A).

In the PLS-DA score plots based on UHPLC-LTQ-Orbitrap-MS data sets, the control, non- gardening, and gardening were clearly distinguished by PLS1 (6.29%) and PLS2 (1.99%) with model parameters including R^2^X (0.204), R^2^Y (0.984), Q^2^ (0.772), and *p*-value (<0.05) (Figure 1B). However, the PCA score plots showed no difference, as observed by PCA of the GC-TOF-MS data sets (Figure S1B).

Significantly different metabolites among experimental groups were selected by the variable importance in the projection (VIP) value (>0.7) of the PLS-DA models and the *p*-value (<0.05) as evaluated by a *t*-test and analysis of variance for statistical significance. The selected metabolites were tentatively identified by comparing their retention times and mass fragment patterns with standard compounds, various parameters such as mass fragment patterns, elemental composition, and delta ppm value and various databases including the Human Metabolome Database (http://www.hmdb.ca/), National Institutes of Standards and Technology library, and Wiley 9. A total of 40 metabolites including three organic acids, nine amino acids, two carbohydrates, five lipids, seven tryptophan-related metabolites, one other, three non-identifications, and 10 lysophosphatidylcholines were putatively found to significantly differ among the experimental groups and the relative levels of metabolites were expressed as fold-changes (Tables S2 and S3).

### 3.4. Metabolic Pathway and Correlation Analysis between Various Metabolite and Bio-Psychosocial Parameter Following Gardening Intervention with Quantification for Validation

**Figure 2:**
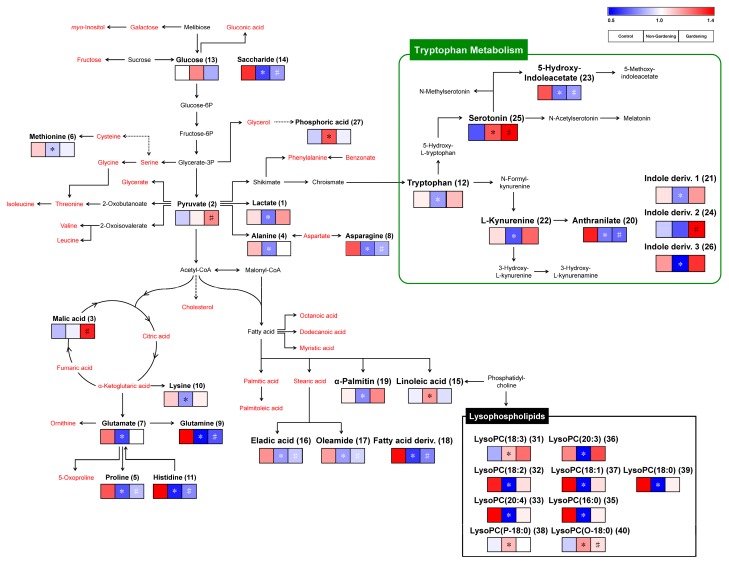
Scheme of the metabolic pathway and relative metabolite levels in the experimental group including control, non-gardening, and gardening subjects. The pathway was modified from the Kyoto Encyclopedia of Genes and Genomes (KEGG) database (http://www.genome.jp/kegg/). The red characters indicate the metabolite detected by Gas chromatography time-of-flight mass spectrometry (GC-TOF-MS) and Ultrahigh-performance liquid chromatography Linear-ion-trap Orbitrap mass spectrometry (UHPLC-LTQ-Orbitrap-MS) with no significance. The colored squares (blue-to-red) represent the relative metabolite abundance in the experimental group including control, non-gardening, and gardening.

Based on our results showing subtle variations in serum metabolites following gardening intervention or not, we observed a metabolic pathway coupled with major variables in our data (Figure 2). The proposed metabolic pathway can be used to understand the relationship between metabolites and horticultural therapy effects. The relative levels of selected metabolites differed between the control group and non-gardening and gardening groups. Most amino acid levels, except for tryptophan, decreased in the non-gardening and gardening groups when compared to the control group. Moreover, fatty acids and lysophospholipids, except for lyso PC (18:3), lyso PC (20:3), lyso PC (P-18:0), and lyso PC (O-18:0), showed similar patterns to the amino acids. However, tryptophan metabolism-related metabolites such as serotonin, kynurenine, and indole derivatives were slightly increased in the gardening group compared to the control group. Especially, serotonin and indole derivative 2 significantly differed between the gardening group and control group (*p* < 0.05). Furthermore, organic acids such as lactic acid, pyruvic acid, and malic acid were increased in the gardening group.

**Figure 3:**
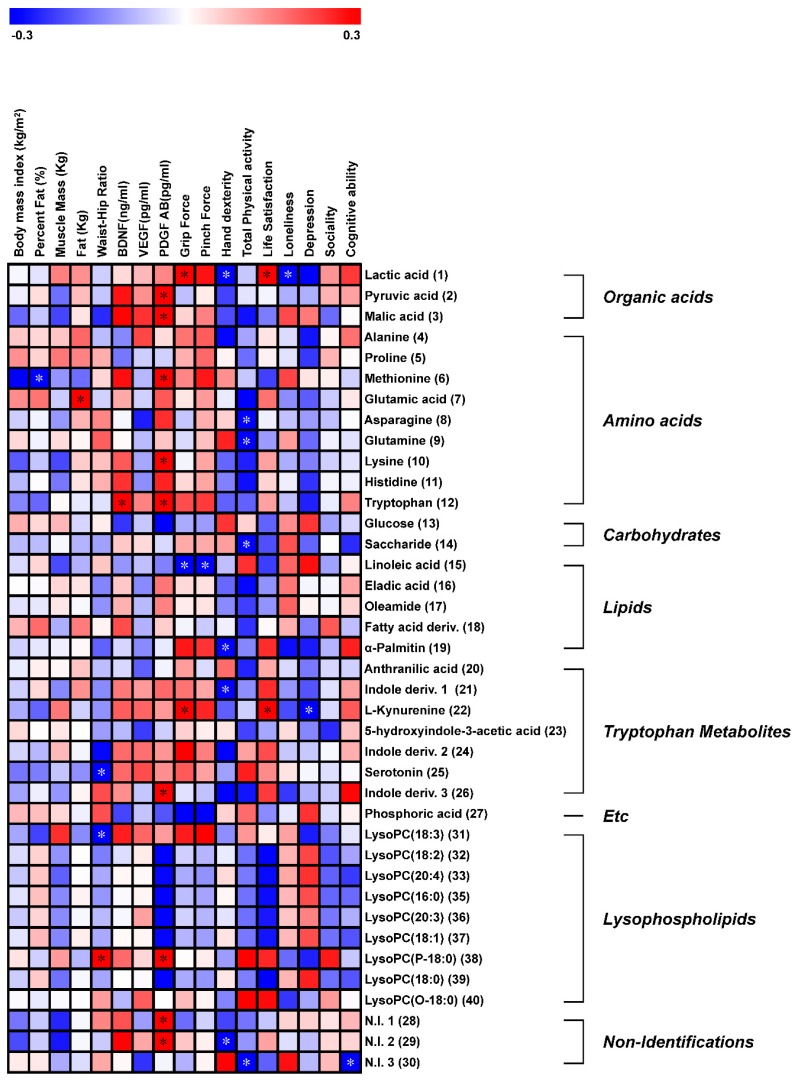
Correlation patterns among metabolites, body composition, growth factor levels, and cortisol (brain-derived neurotrophic factor (BDNF), vascular endothelial growth factor (VEGF), and platelet-derived growth factor (PDGF)), hand function ability, physical activity, and a psychological factor. Each square indicates the Pearson’s correlation coefficient values (*r*). The red and blue colors represent positive (0 < *r* < 0.3) and negative (−0.3 < *r* < 0) correlations, respectively. * *p*-value < 0.05.

Moreover, we performed correlation analysis between different metabolites and various bio-psychosocial parameters (Figure 3). Among them, several tryptophan metabolism-related metabolites such as tryptophan, kynurenine, serotonin, and indole derivatives, which were slightly increased in the gardening groups compared to in the control, showed a positive correlation with growth factor levels and cortisol (BDNF, VEGF, and PDGF) and psychological factors (life satisfaction and cognitive ability), while loneliness and depression were negatively correlated. Additionally, organic acids (lactic acid, pyruvic acid, and malic acid), which were slightly increased in the gardening group when compared to the control group, were negatively correlated with hand dexterity.

Based on the metabolic pathway and correlation analysis, we selected four metabolites for validation, which showed significant correlation coefficients (*p* < 0.05) and were slightly increased in the gardening group compared to the control group (Table 6). Four selected metabolites were quantified in the control, non-gardening, and gardening subjects. As a result, the serum serotonin concentrations in the control, non-gardening, and gardening subjects were 1.21 ± 0.93, 2.37 ± 1.75, and 2.61 ± 1.54 μg/mL, respectively (*p* = 0.001, by using the *t*-test between the control and gardening subjects). The remaining metabolites such as lactic acid, tryptophan, and kynurenine were increased in gardening subjects compared to the controls, but the differences were not significant. The levels of the selected metabolites were also consistent with the results of metabolites analysis.

## 4. Discussion

In this study, we investigated the effects of gardening intervention in older adults using metabolomic approaches. Gardening intervention is emerging as an increasingly prevalent non-pharmacological approach for improving the quality of life of older adults. This gardening intervention has beneficial effects on cognitive functions, psychology, and physical activity, including preventing cognitive decline and depression, encouraging social integration, and improving health conditions [52]. However, metabolomic approaches for a gardening intervention have not been widely used. In metabolomic analysis of the control, non-gardening, and gardening subjects, different patterns and bio-psychosocial parameters such as growth factor levels and cortisol, hand function ability, and psychological factors among experimental groups were observed. To evaluate the differences in metabolites, we carried out MS-based non-targeted analysis in the control, non- gardening, and gardening subjects.

Serum metabolomics showed that the gardening group had slightly increased organic acids, tryptophan, and tryptophan-related metabolites compared to the control group. Particularly, pyruvic acid, malic acid, serotonin, and indole derivatives differed significantly between the control and gardening subjects (Table S2 and Figure 3). Generally, tryptophan is an important amino acid that is the precursor of various physiologically essential metabolites. These metabolites are catabolized through two major pathways including the methoxyindole pathway and the kynurenine pathway [53]. Abnormal conditions related to central nervous system (CNS) disease, including a wide range of pathophysiologies and even psychiatric disorders such as schizophrenia and depression, are associated with tryptophan metabolism [53]. Among tryptophan metabolism-related metabolites, the level of kynurenine was decreased in patients with depression compared to healthy subjects. However, the role of serum kynurenine in depression remains unclear [54]. Moreover, the contents of kynurenine-related metabolites correspond with the elevated plasma levels of IL-6, which is the predominant marker of leukoaraiosis and influences cerebral infarction in patients with depression, while IL-6 was significantly decreased in subjects who underwent a gardening intervention [52,54]. Serotonin plays an important role as a neurotransmitter and prominent role in brain development including cognition, emotion, and pain sensitivity. Psychological abnormalities in depression and emotional expression have been linked to serotonin levels, which showed a decreasing pattern [55]. Recent research discovered that antidepressants that work on serotonin receptors increased the hippocampal BDNF level and of BDNF positive neurons as compared to a placebo [56]. This finding may suggest that gardening has similar biological effects as antidepressants. Organic acid such as lactic acid, pyruvic acid, and malic acid were increased in the gardening group compared to the controls. Pyruvic acid, which is involved in the glycolysis process, is catabolized from glucose and then passed into the tricarboxylic acid cycle to produce ATP [57]. This metabolite is an important energy source in the brain and circulation system, maintained the improvements in cognitive function, and improved neuron survival [58]. Lactic acid, which is the end-product of anaerobic energy metabolism, is a strong acid that breaks down into lactate and hydrogen ions [59]. Lactic acid has been suggested an important cause of muscle fatigue and has little effect on muscle contraction [58]. In this study, variations in the levels of organic acids, tryptophan metabolism-related metabolites such as malic acid, pyruvic acid, kynurenine, serotonin, and indole derivatives were significantly increased by a gardening intervention. These results may be helpful for explaining the effectiveness of gardening intervention on pathophysiologies, psychiatric disorders, and muscle ability. Several previous studies of CNS-related diseases revealed significant correlations between tryptophan metabolism-related metabolites and CNS-linked parameters such as growth factor levels, cortisol, and psychological factors [54,60,61,62]. Additionally, impairments in memory, cognition, and muscle ability were shown to be correlated with pyruvate and lactic acid [58,59]. In our correlation analysis (Figure 3), most organic acids and tryptophan metabolism-related metabolites were correlated with growth factor levels and cortisol (BDNF, VEGF, and PDGF), psychological factors (life satisfaction, depression, and cognitive ability), and hand function ability (grip force, pinch force, and hand dexterity). Collectively, gardening intervention increased organic acid levels and tryptophan metabolism and can contribute to improving CNS-related conditions and muscle ability. However, a small sample size, convenience sampling, and non-randomization of the samples in the study design do not allow fair representation of the samples and make external validity difficult to maintain. These remain as a limitation of the study, deterring to draw solid generalized conclusions.

## 5. Conclusions

The 24-session gardening program in this study significantly improved cognitive function and BDNF levels related to cognitive health factors of the elderly participating. In addition, we identified serum tryptophan, kynurenine, and serotonin as the bio-makers for improved cognitive ability by gardening activity using metabolomic analysis. These results suggest that gardening could be used as a physical activity for maintaining or improving cognitive health in the elderly. We also provided scientific evidence of the fundamental mechanism that causes the cognitive effects in the elderly through gardening activity. This study contributes to a better understanding of the mechanism of gardening activities.

Future studies should explore the application of gardening activities to broader subjects for cognitive health. In such attempts, assessment of therapeutic or preventative effects by gardening activities on elderly people with dementia or cognitively disabled individuals will be focused on in future studies.

## Figures and Tables

**Table 1:** A 24-session gardening program for the improvement of cognitive function of the elderly.

Session	Gardening Activity	Plant Used	Gardening Tool	Estimated METs ^1^
1	Design garden and making garden plot	-	Shovel, rake, base fertilizer	4
2	Planting transplants	Lettuce (*Lactuca sativa* L.)	Trowel, watering can	3.5
		Radish (*Raphanus sativus*)		
3	Making medicated plant garden beds	Siler divaricate (*Ledebouriella seseloides*)	Trowel, watering can	3.5
		Korean angelica (*Angelica gigas*)		
4	Planting transplants	Pepper (*Capsicum annuum*)	Trowel, additional fertilizer, watering can	4
		Eggplant (*Solanum melongena*)		
		Broccoli (*Brassica oleracea*)		
5	Planting transplants	Tomato (*Solanum lycopersicum* L.)	Trowel, watering can	2.8
		Chives (*Allium tuberosum*)		
6	Planting transplants	Bean (*Phaseolus vulgaris* L.)	Trowel, watering can	2.7
7	Making vegetable garden beds	Lettuce (*Lactuca sativa*)	Gardening box, trowel, watering can, peat-moss, perlite	2.8
		Chinese cabbage (*Brassica rapa subsp. pekinensis*)		
8	Making vegetable garden beds	Oak leaf (*Lactuca sativa*)	Gardening box, trowel, watering can, peat-moss, perlite	2.9
9	Planting transplants	Paprika (*Capsicum annuum*)	Trowel, watering can	2.8
		Cucumber Pepper (*Capsicum annuum*)		
10	Making organic fertilizers	-	Egg, vinegar, plastic bottle, watering can	2.5
11	Making medicinal plants garden beds	Deodeok (*Codonopsis lanceolate*)	Shovel, rake, hoe, trowel, watering can	3.5
12	Planting transplants	Sweet potato (*Ipomoea batatas*)	Spray, organic fertilizers, hoe, trowel, watering can	3
13	Planting transplants	Perilla (*Perilla frutescens*)	Stick, tie, trowel, watering can	2.8
14	Making flower garden beds	Brazilian jasmine (*Mandevilla sanderi*)	Gardening box, trowel, hoe, watering can, peat-moss, perlite	2.7
		Italian Aster (*Aster amellus*)		
		Lobelia (*Lobelia erinus*)		
15	Maintaining garden	-	Spray, organic fertilizers, hoe, trowel, watering can	3.5
16	Making flower garden beds	Cockscomb (*Celosia cristata*)	Shovel, rake, hoe, trowel, watering can	4
		African marigold (*Tagetes erecta*)		
		Rose moss (*Portulaca grandiflora*)		
17	Making herb garden beds	Rosemary (*Rosmarinus officinalis*)	Shovel, rake, hoe, trowel, watering can	4
		Choco mint (*Menthax piperita*)		
18	Hydroponics	Golden Pothos (*Epipremnum aureum*)	Hydroball, pot, bucket, watering can	2.5
		Peace lily (*Spathiphyllum*)		
19	Planting transplants	Spring onion (*Allium fistulosum*)	Trowel, watering can	2.8
20	Sowing seeds	Lettuce (*Lactuca sativa*)	Tray, trowel, peat-moss, perlite, watering can	2.8
21	Cutting stems	Rosemary (*Rosmarinus officinalis*)	Pot, scissors, peat-moss, perlite	2.7
		Spearmint (*Mentha spicata*)		
		Lavender (*Lavandula sp*.)		
22	Making medicated plant garden beds	Bean (*Glycine max*)	Shovel, rake, hoe, trowel, watering can	4
		Yacon (*Polymnia sonchifolia*)		
23	Harvesting and packing harvests	-	-	2.8
24	Garden party	-	-	2.7

^1^ Estimated metabolic equivalents (METs) based on the previous studies for measuring exercise intensities of gardening tasks [22,42] and a study for a compendium of physical activities [43]. Intensities below 3.0 METs indicate a low-intensity physical activity and above 3.0 to 6.0 METs presents moderate-intensity physical activities.

**Table 2:** Comparisons of demographic information of the subjects in a study by using chi-square and an independent *t*-test ^1^.

Variable	Gardening (N = 20)	Non-Gardening (N = 20)	*p* ^2^
Age (years)	71.8 ± 4.8 ^3^	75.9 ± 5.3	0.017 *
Size of a family	2.6 ± 1.4	2.9 ± 1.6	0.607 NS
Body composition ^4^			
Height (cm)	158.0 ± 8.3	153.7 ± 8.5	0.199 NS
Body weight (kg)	58.6 ± 6.5	56.3 ± 9.6	0.521 NS
Lean mass (kg)	37.3 ± 5.2	35.8 ± 6.5	0.161 NS
Fat (kg)	18.2 ± 4.7	18.3 ± 4.6	0.932 NS
Body mass index (kg·m-2)	23.8 ± 2.7	24.3 ± 2.8	0.535 NS
Percent fat (%)	30.9 ± 6.9	31.8 ± 6.2	0.839 NS
Physical activity (MET-min/week) ^5^			
Walking	986.5 ± 988.5	1330.4 ± 1029.3	0.490 NS
Moderate	1084.2 ± 1240.7	1524.2 ± 1355.6	0.147 NS
Vigorous	454.7 ± 1343.2	252.6 ± 722.1	0.378 NS
Total	2525.5 ± 2192.8	3107.3 ± 1492.6	0.382 NS
Gender			0.047 *
Male	10 (50.0)	4 (20.0)	
Female	10 (50.0)	16 (80.0)	
Disease			
Hypertension	8 (40.0)	10 (50.0)	0.723 NS
Hyperlipidaemic	6 (35.0)	5 (25.0)	0.525 NS
Arthritis	2 (10.0)	7 (35.0)	0.058 NS
Diabetes	2 (10.0)	2 (10.0)	0.100 NS
Gastritis	1 (5.0)	3 (15.0)	0.292 NS
Benign prostatic hyperplasia	2 (10.0)	1 (5.0)	0.472 NS
Cardiac disorder	1 (5.0)	1 (5.0)	0.100 NS
Depression	1 (5.0)	1 (5.0)	0.100 NS
Cupulolithiasis	0 (0.0)	1 (5.0)	0.311 NS
Glaucoma	1 (5.0)	0 (0.0)	0.311 NS
Fatty liver	1 (5.0)	0 (0.0)	0.311 NS
Osteoporosis	1 (5.0)	0 (0.0)	0.311 NS
Anemia	0 (0.0)	1 (5.0)	0.311 NS
Education			0.018 *
Elementary school graduate or less	2 (10.0)	3 (15.0)	
Middle school graduate	2 (10.0)	12 (60.0)	
High school graduate	11 (55.0)	4 (20.0)	
College graduate	1 (5.0)	0 (0.0)	
University graduate	3 (15.0)	1 (5.0)	
Graduate school graduate	1 (5.0)	0 (0.0)	
Marital status			0.856 NS
Married	13 (65.0)	12 (60.0)	
Widowed	4 (20.0)	6 (30.0)	
Divorced	2 (10.0)	1 (5.0)	
Unmarried	1 (5.0)	5 (5.0)	

^1^ Chi-square was used to compare values at *p* = 0.05 for disease, education, marital status, monthly income, and an independent *t*-test was used to compare means at *p* = 0.05 for the age, size of a family, body composition, and physical activity. ^2^ * Significant at *p* < 0.05 by using chi-square or an independent *t*-test between gardening and non-gardening group. ^3^ Data presented as mean ± standard deviation or *n* (%). ^4^ Measured using a body fat analyzer (ioi 353). ^5^ IPAQ-SF is self-reported physical activity measured in metabolic equivalent of task (MET) minutes. A MET-minute is computed by multiplying the MET score by the minutes performed [49]. Walking MET-min/week = 3.3 × walking min × walking days. Moderate MET-min/week = 4.0 × moderate-intensity activity min × moderate days. Vigorous MET-min/week = 8.0 × vigorous-intensity activity min × vigorous-intensity days. A combined total physical activity MET-min/week can be computed as the sum of Walking + Moderate + Vigorous MET-min/week scores [49].

**Table 3:** Comparisons of cognitive ability between gardening intervention and control groups in the elderly by using an independent *t*-test (Mean ± SD).

Mini Mental State Examination (K-MMES) ^1^	Group		*p* ^2^
	Gardening (N = 20)	Non-Gardening (N = 20)	
Pre-test	27.7 ± 2.1	26.4 ± 2.3	0.083 NS
Post-test	28.3 ± 1.6	26.3 ± 2.3	0.003 **

^1^ K-MMSE: 0–17 = severe cognitive impairment. 18–23 = mild cognitive impairment. 24–30 = no cognitive impairment [48]. ^2^ ** and NS Significant at *p* < 0.01 and non-significant at *p* < 0.05.

**Table 4:** Comparisons of hand function ability before and after gardening intervention in the elderly by using a paired *t*-test (Mean ± SD).

Hand Function		Group	
		Gardening (N = 20)	Non-Gardening (N = 20)
Grip force (kg)	Pre-test	28.2 ± 9.7	23.2 ± 7.5
	Post-test	27.0 ± 9.1	22.5 ± 8.8
	*p* ^1^	0.097 NS	0.293 NS
Pinch force (kg)	Pre-test	13.1 ± 3.4	11.4 ± 4.8
	Post-test	13.0 ± 3.5	11.0 ± 4.7
	*p*	0.922 NS	0.385 NS
Hand dexterity (s)	Pre-test	80.6 ± 15.2	96.3 ± 18.3
	Post-test	76.9 ± 13.6	98.6 ± 22.6
	*p*	0.024 *	0.508 NS

^1^ NS and * Non-significant or significant at *p* < 0.05 by using a paired *t*-test on the variables between the pre-test and post-test in each group.

**Table 5:** Comparisons of the senior fitness test before and after gardening intervention in the elderly by using a paired *t*-test (Mean ± SD).

SFT		Group	
		Gardening (N = 20)	Non-Gardening (N = 20)
Chair stand (n)	Pre-test	19.1 ± 3.4	17.2 ± 4.0
	Post-test	20.3 ± 5.7	18.3 ± 6.6
	*p*	0.559 NS	0.090 NS
Arm curl (n)	Pre-test	19.1 ± 3.4	18.4 ± 4.4
	Post-test	20.7 ± 5.7	19.9 ± 5.6
	*p*	0.238 NS	0.270 NS
2-Minute Step Test (n)	Pre-test	100.9 ± 14.1	94.9 ± 15.2
	Post-test	102.2 ± 15.8	99.7 ± 26.5
	*p*	0.606 NS	0.221 NS
2.45-m up-and-go (s)	Pre-test	5.1 ± 0.9	5.9 ± 1.3
	Post-test	4.7 ± 1.0	5.9 ± 1.1
	*p*	0.582 NS	0.810 NS
Chair-sit-and-reach (cm)	Pre-test	22.1 ± 6.1	23.3 ± 9.4
	Post-test	24.0 ± 7.6	21.4 ± 11.0
	*p*	0.141 NS	0.267 NS
Back scratch (cm)	Pre-test	−5.8 ± 15.2	−9.2 ± 12.6
	Post-test	−5.3 ± 10.0	−14.9 ± 27.4
	*p*	0.312 NS	0.270 NS

**Table 6:** Mean concentrations of potential metabolite distinguishing experimental groups including control, non-gardening, and gardening subjects as quantified by Gas chromatography time-of-flight mass spectrometry (GC-TOF-MS).

No.	Metabolites ^a^	Mean Concentration (μg/100 μL Serum)			Fold Change	
		Control	Non-Gardening	Gardening	Non-gardening/Control	Gardening/Control
1	Lactic acid	0.78 ± 0.30	0.49 ± 0.29 *	0.86 ± 0.30	**0.63**	**1.10**
2	Tryptophan	0.23 ± 0.06	0.19 ± 0.05 *	0.24 ± 0.04	**0.82**	**1.02**
3	Kynurenine	0.37 ± 0.20	0.23 ± 0.14 *	0.42 ± 0.19	**0.63**	**1.13**
4	Serotonin	0.12 ± 0.09	0.24 ± 0.17 *	0.26 ± 0.15 ^#^	**1.96**	**2.15**

The color scheme is as follows: lower limit value, 0.6 (blue), middle value, 1.0 (white), upper limit value, 1.3 (red).^a^ Four selected metabolites were represented using a heat map with fold change (Non-gardening/Control, Gardening/Control) indicated by heat scale * *p*-value < 0.05 by using the *t*-test between control and non-gardening groups. The ^#^ *p*-value < 0.05 when using the *t*-test between control and gardening groups.

## Supplementary Materials

Supplementary Materials can be found at https://www.mdpi.com/1660-4601/17/2/541/s1. Figure S1: Principal component analysis (PCA) (A, B) score plots of the serum sample from control, non-gardening, and gardening group using data from GC-TOF-MS (A) and UHPLC-Orbitrap-MS (B) analysis. Table S1: Effects of a gardening activity program on brain nerve growth factor levels in the elderly by using paired *t*-test (Mean ± SD). Table S2: Significantly differentiating metabolites between experimental group including control, non-gardening, gardening subjects analyzed by GC-TOF-MS. Table S3: Significantly differentiating metabolites between experimental group including control, non-gardening, gardening subjects analyzed by UHPLC-LTQ-Orbitrap-MS.
