# Supplementary material for: Metabolite Profiling Revealed That a Gardening Activity Program Improves Cognitive Ability Correlated with BDNF Levels and Serotonin Metabolism in the Elderly
Source: Int J Environ Res Public Health. 2020 Jan 15;17(2):541. doi: 10.3390/ijerph17020541 (PMC7014360; doi:10.3390/ijerph17020541)
Supplement: Supplementary file 1 [file ijerph-17-00541-s001.pdf]

Supplementary Figure

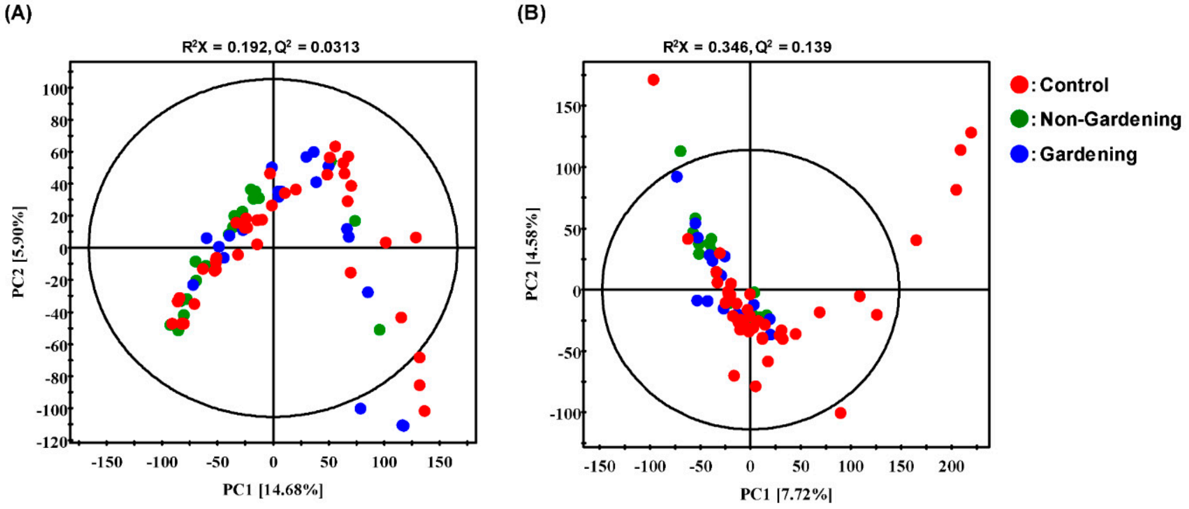

Figure S1. Principal component analysis (PCA) (A, B) score plots of the serum sample from control, non-gardening, and gardening group using data from GC-TOF-MS (A) and UHPLC-Orbitrap-MS (B) analysis. ● Control, ● Non-gardening, ● Gardening

Supplementary Tables

Table S1 Effects of a gardening activity program on brain nerve growth factor levels in the elderly by using paired *t*-test (Mean ± SD).

**Variable**		**Group**	
		**Gardening (N = 20)**	**Non-gardening (N = 20)**
BDNF (ng/ml)	Pre-test	25.0 ± 12.5	29.4 ± 10.1
	Post-test	29.7 ± 17.6	24.7 ± 5.9
	P[1]	0.047 *	0.047 *
PDGF (pg/ml)	Pre-test	3332.1 ± 2058.6	2943.0 ± 1599.0
	Post-test	3463.9 ± 2641.1	2486.6 ± 1160.8
	P	0.686 NS	0.128 NS

[1] NS and * non-Significant or significant at $p < 0.05$ by using paired *t*-test on the variables between pre-test and post-test in each group.

Table S2 Significantly differentiating metabolites between experimental group including control, non-gardening, gardening subjects analyzed by GC-TOF-MS

No.	Ret [a]	VIP1	VIP2	Unique Mass (*m*/*z*)	Metabolites [b]	Fold Change [c]			MS Fragment pattern (*m*/*z*)	TMS [d]	ID [e]
						Control	Non-gardening	Gardening			
Organic acids											
1	5.10	0.54	1.69	191	Lactic acid	1.07	0.69*	1.16	73, 117, 147, 75, 66, 191, 59, 74	2TMS	STD/MS
2	5.94	1.56	1.91	88	Pyruvic acid	0.88	1.05	1.18#	73, 147, 133, 59, 86, 100, 220, 89	2TMS	STD/MS
3	9.16	1.14	1.63	147	Malic acid	0.85	0.97	1.33#	73, 147, 75, 133, 55, 233, 74, 59, 148	3TMS	STD/MS
Amino acids											
4	5.53	1.86	1.37	116	Alanine	1.13	0.72*	1.02	116, 73, 147, 59, 117, 75, 148, 74	1TMS	STD/MS
5	7.49	2.65	2.03	142	Proline	1.27	0.62*	0.84#	142, 73, 143, 70, 147, 59, 144, 216	2TMS	STD/MS
6	9.43	1.83	1.40	176	Methionine	1.09	0.87*	0.95	73, 176, 128, 61, 147, 75, 100, 59	2TMS	STD/MS
7	10.21	3.10	2.21	246	Glutamic acid	1.22	0.57*	1.00#	73, 246, 75, 147, 128, 84, 100, 156	3TMS	STD/MS
8	10.63	2.90	2.14	116	Asparagine	1.26	0.67*	0.82#	73, 116, 75, 132, 231, 141, 188, 100	3TMS	STD/MS
9	11.38	4.10	3.21	156	Glutamine	1.48	0.44*	0.60#	73, 156, 75, 218, 226, 147, 100, 74	3TMS	STD/MS
10	12.41	1.91	1.56	156	Lysine	1.09	0.77*	1.04	73, 147, 75, 156, 205, 174, 103, 74	4TMS	STD/MS
11	12.45	2.56	1.83	154	Histidine	1.39	0.49*	0.73#	73, 154, 75, 147, 205, 74, 103, 217	3TMS	STD/MS
12	14.33	1.83	1.66	202	Tryptophan	1.06	0.78*	1.10	202, 73, 203, 75, 291, 204, 117, 129, 131	3TMS	STD/MS
Carbohyrates											
13	12.35	0.55	1.73	160	Glucose	1.01	1.20	0.79	73, 147, 160, 205, 103, 129, 217, 319	5TMS	STD/MS
14	12.71	3.65	2.63	217	Saccharide	1.33	0.59*	0.76#	73, 217, 147, 75, 103, 156, 129, 74, 157	5TMS	MS
Lipids											
15	14.14	1.62	1.23	150	Linoleic acid	0.95	1.18*	0.93	75, 73, 67, 81, 129, 117, 95, 82, 337	1TMS	STD/MS
16	14.20	2.70	1.93	144	Eladic acid	1.18	0.77*	0.87#	75, 73, 117, 129, 131, 145, 144, 339	1TMS	STD/MS
17	15.29	2.77	1.97	144	Oleamide	1.16	0.77*	0.91#	73, 131, 144, 116, 128, 115, 198, 338	1TMS	STD/MS
18	15.54	3.15	2.28	91	Fatty acid deriv.	1.37	0.54*	0.71#	79, 91, 67, 77, 105, 117, 119, 80, 92	1TMS	MS
19	16.19	1.18	1.27	371	α–Palmitin	1.04	0.73*	1.19	73, 147, 57, 55, 75, 129, 203, 371, 103	2TMS	STD/MS
Tryptophan Metabolites											
20	12.77	1.93	1.51	266	Anthranilic acid	1.34	0.67*	0.65#	73, 266, 147, 237, 217, 209, 135, 165	3TMS	MS
21	13.89	1.05	1.43	202	Indole deriv. 1	1.06	0.71*	1.17	73, 147, 202, 75, 221, 250, 207	–	MS
22	14.08	1.07	1.39	202	L-Kynurenine	1.08	0.60*	1.25	73, 202, 131, 170, 231, 267, 319	3TMS	MS
23	14.26	2.59	2.12	202	5-hydroxyindole-3-acetic acid	1.27	0.69*	0.77	73, 202, 131, 117, 129, 116, 341, 219	3TMS	MS
24	14.48	0.11	1.38	202	Indole deriv. 2	0.86	0.62	1.66#	73, 202, 118, 172, 276, 334, 373	–	MS
25	15.69	3.75	3.21	202	Serotonin	0.61	1.31*	1.46#	73, 202, 129, 55, 147, 100, 131	3TMS	STD/MS
26	16.11	1.93	1.71	202	Indole deriv. 3	1.19	0.37*	1.25	73, 147, 129, 202, 67, 91, 103, 203	–	MS
Etc.											

27	7.30	2.83	2.16	299	Phosphoric acid	0.88	1.27*	0.97	73, 299, 133, 211, 300, 74, 193, 135	2TMS	STD/MS
Non-identifications											
28	7.84	0.86	0.99	184	N.I. 1	0.92	0.93	1.24#	73, 184, 134, 59, 77, 86, 100, 69	–	–
29	8.92	0.19	1.78	350	N.I. 2	0.90	0.57	1.63#	73, 147, 59, 350, 75, 133, 131, 128	–	–
30	9.33	3.78	3.07	180	N.I. 3	1.37	0.61*	0.65#	180, 75, 110, 73, 58, 71, 69, 59	–	–

Metabolites selected by VIP > 0.7 *p*-value < 0.05 from PLS-DA model; [a] Retention time; [b] Tentatively identified metabolites by using HMDB, NIST, and in-house library; [c] Relative level of metabolites were normalized by the mean values of each sets; [d] Trimethylsilyl; [e] Identification, STD-commercial standard compound, MS-comparison with the mass spectra; * *p*-value < 0.05 by *t*-test between Control and Non-gardening groups. # *p*-value < 0.05 by *t*-test between Control and Gardening groups.

Table S3. Significantly differentiating metabolites between experimental group including control, non-gardening, gardening subjects analyzed by UHPLC-LTQ-Orbitrap-MS

No.	Ret [a]	Identifed ion (*m/z*)	Adduct	Tentative Metabolites [b]	Fold change [c]			Molecular formula	PPM	MS^n Fragment pattern (*m/z*) [d]	ID [e]
					Control	Non-gardening	Gardening				
Lysophospholipids											
31	8.14	518.3225	$[M+H]^+$	LysoPC(18:3)	0.84	1.10*	1.23	C26H48NO7P	-3.0	n.d.	HMDB
32	8.52	520.3378	$[M+H]^+$	LysoPC(18:2)	1.33	0.27*	1.07	C26H50NO7P	-3.7	520> 502> 443> 184	HMDB
33	8.54	544.3376	$[M+H]^+$	LysoPC(20:4)	1.39	0.18*	1.04	C28H50NO7P	-4.7	544> 526> 467> 184	HMDB
34	8.71	496.3378	$[M+H]^+$	LysoPC(16:0) [f]	1.38	0.21*	1.02	C24H50NO7P	-3.7	496> 478> 184, 166, 124	HMDB
35	8.83	496.3378	$[M+H]^+$	LysoPC(16:0) [f]	1.43	0.15*	0.98	C24H50NO7P	-3.7	496> 478> 184, 166, 124	HMDB
36	8.83	546.3760	$[M+H]^+$	LysoPC(20:3)	1.25	0.21*	1.28	C28H52NO7P	-4.9	546> 528> 469> 184	HMDB
37	9.04	522.3536	$[M+H]^+$	LysoPC(18:1)	1.41	0.16*	1.02	C26H52NO7P	-4.3	522> 504> 445> 184	HMDB
38	9.31	508.3744	$[M+H]^+$	LysoPC(P-18:0)	0.95	1.11*	0.98	C26H54NO6P	-4.7	580> 490, 431, 240, 184	HMDB
39	9.71	524.3693	$[M+H]^+$	LysoPC(18:0)	1.40	0.19*	1.01	C26H54NO7P	-4.5	524> 506, 447> 311, 184	HMDB
40	10.06	510.3896	$[M+H]^+$	LysoPC(O-18:0)	0.89	1.16*	1.60#	C26H56NO6P	-4.2	510> 492, 433, 240, 184	HMDB

Metabolites selected by VIP > 0.7 from PLS-DA model; LysoPC, lysophosphatidylcholine; n.d., Not detected; [a] Retention time; [b] Tentatively identified metabolites by using HMDB: The Human Metabolome Data Base (http://hmdb.ca/), NIST, PPM (high resolution) and Reference (Suh *et al*., 2017 [625159]); [c] Relative level of metabolites were normalized by the mean values of each sets; [d] MS^n fragment patterns detected in positive ion mode.; [e] This means the 2 forms of LysoPC with the fatty acyl groups at *sn*-1 or *sn*-2 on the glycerol backbone; *p-value < 0.05 by t-test between Control and Non-gardening groups. #p-value < 0.05 by t-test between Control and Gardening groups.
